# miR-15a-3p Protects Against Isoniazid-Induced Liver Injury *via* Suppressing *N*-Acetyltransferase 2 Expression

**DOI:** 10.3389/fmolb.2021.752072

**Published:** 2021-11-23

**Authors:** Xinmei Li, Heng Zhang, Lin Xu, Yuan Jin, Jiao Luo, Chuanhai Li, Kunming Zhao, Yuxin Zheng, Dianke Yu, Yanjie Zhao

**Affiliations:** School of Public Health, Qingdao University, Qingdao, China

**Keywords:** isoniazid, drug-induced liver injury, N-acetyltransferase 2, hsa-miR-15a-3p, regulation

## Abstract

Isoniazid (INH), an effective first-line drug for tuberculosis treatment, has been reported to be associated with hepatotoxicity for decades, but the underlying mechanisms are poorly understood. *N*-acetyltransferase 2 (NAT2) is a Phase II enzyme that specifically catalyzes the acetylation of INH, and NAT2 expression/activity play pivotal roles in INH metabolism, drug efficacy, and toxicity. In this study, we systematically investigated the regulatory roles of microRNA (miRNA) in *NAT2* expression and INH-induced liver injury via a series of *in silico*, *in vitro*, and *in vivo* analyses. Four mature miRNAs, including hsa-miR-15a-3p, hsa-miR-628-5p, hsa-miR-1262, and hsa-miR-3132, were predicted to target the *NAT2* transcript, and a negative correlation was observed between hsa-miR-15a-3p and *NAT2* transcripts in liver samples. Further experiments serially revealed that hsa-miR-15a-3p was able to interact with the 3′-untranslated region (UTR) of *NAT2* directly, suppressed the endogenous *NAT2* expression, and then inhibited INH-induced *NAT2* overexpression as well as INH-induced liver injury, both in liver cells and mouse model. In summary, our results identified hsa-miR-15a-3p as a novel epigenetic factor modulating *NAT2* expression and as a protective module against INH-induced liver injury, and provided new clues to elucidate the epigenetic regulatory mechanisms concerning drug-induced liver injury (DILI).

## Introduction

Drug-induced liver injury (DILI) refers to the liver impairment caused by naturally existing or manufactured hepatotoxic substances, which enter the liver for biotransformation mainly through gastrointestinal tract and blood circulation, and their metabolites or themselves make the liver suffering from varying degrees of damage (Fontana, 2014; Tolosa et al., 2018). In China, the incidence of DILI is 3.80/100,000 per year, and traditional Chinese medicine, herbs and dietary supplements, and anti-tuberculosis drugs are considered as the main causes of DILI (Shen et al., 2019).

Isoniazid (INH), one main first-line anti-tuberculosis drug, was reported to be associated with DILI since its introduction into the market in 1952. (Boelsterli and Lee, 2014). Tuberculosis is caused by the *Mycobacterium tuberculosis* that could transmit among humans *via* the respiratory route. As one of the ancient human diseases, tuberculosis is still one of the leading causes of death among all infectious diseases (Bloom et al., 2017; Natarajan et al., 2020), but clinical treatment is usually accompanied by a high incidence of INH-induced liver injury.

Most chemicals or drugs are substrates for diverse drug metabolizing enzymes (DMEs) in liver, including Phase I enzymes such as Cytochrome P450s, and Phase II enzymes such as UDP-glucuronosyltransferases (UGTs), glutathione-*S*-transferases (GSTs), and *N*-Acetyltransferases (NATs). The metabolism of INH in liver has been well-documented by several reports (Metushi et al., 2016; Erwin et al., 2019). Briefly, INH is metabolized by *N*-acetyltransferase 2 (NAT2) to produce acetyl-INH, most acetyl-INH is excreted and the remaining can be hydrolyzed to acetylhydrazine (AcHz), and then the AcHz is catalyzed either by *NAT2* to produce nontoxic diacetyl-hydrazine (DiAcHz), or by *CYP2E1* to produce multiple hepatotoxins such as acetyldaizene and ketene (Erwin et al., 2019). Eventually, the intermediates formed by *CYP2E1* are detoxified by GSTs. The general pathogenesis of DILI was considered to be initiated by an imbalance between chemical metabolism activation and biological detoxification process (Gu and Manautou, 2012); however, the underlying mechanism of INH-induced liver injury remains unclear.

NATs are responsible for the acetylation of chemicals, which catalyzes the transfer of acetyl group from acetyl-Coenzyme A to form acetylated derivatives (Huang, 2014; Perwitasari et al., 2015; Yew et al., 2018). NAT1 and NAT2 are two major isozymes exhibiting distinct substrate specificities. *NAT1* is responsible for the acetylation of *p*-aminosalicylate and *p*-aminobenzoylglutamate, while *NAT2* acetylates hydralazine, procainamide, isoniazid, and carcinogenic aromatic amines (Sim, 2002; Mitchell, 2020).

Genetic factors were identified to affect NAT2 activities and INH-induced liver injury. For example, the minor alleles of R64W and D122N significantly decreased NAT2 activities, and partly contributed to “slow acetylator phenotype”, which showed a relative slower acetylation rate for INH (Boukouvala and Fakis, 2005). Slow acetylator phenotype was associated with the increased risk of INH-induced liver injury in some reports (Wattanapokayakit et al., 2016; Yuliwulandari et al., 2019), but not in others (Neill et al., 1990; Hwang et al., 1997). These contradictory results suggested that genetic variants fail to fully explain the inter-individual variation of NAT2 activity and personal chance of INH-induced liver injury.

MicroRNA (miRNA), a class of non-coding single-stranded RNA molecules, has been identified as an important epigenetic factor involved in gene expression and diverse biological processes. Commonly, miRNA is able to interfere with protein translation or degrade the mRNAs, by binding to the 3′-UTR of target transcript (Ambros, 2001; Collins and Cheng, 2006; McGill and Jaeschke, 2015). Numerous studies proved the regulatory role of miRNA in DME expression, drug metabolism, and DILI. For example, *CYP3A4* and *CYP1B1* were reported to be modulated by miR-27b, respectively (Tsuchiya et al., 2006; Pan et al., 2009). Our previous studies have identified multiple miRNAs modulating the expression of DME genes in liver, including hsa-miR-29a-3p targeting *CYP2C19*, *ALDH5A1*, and *SLC22A7*, hsa-miR-25-3p targeting *CYP2B6*, hsa-miR-214-3p targeting *CYP2E1*, and hsa-miR-1301-3p targeting *ADH6*, *ALDH5A1*, and *ALDH8A1* (Yu et al., 2015a; Yu et al., 2015b; Jin et al., 2016; Wang et al., 2017; Wang et al., 2020). Further, we identified multiple miRNAs as protective modules in acetaminophen-induced liver injury (Yu et al., 2018). So far, the regulatory mechanisms of non-coding RNAs in *NAT2* expression and INH-induced liver injury is yet unknown.

In our study, we systematically screened miRNA targeting *NAT2* by *in silico* analysis, and predicted and validated the interaction between hsa-miR-15a-3p and *NAT2* 3′-UTR by a series of biochemical assays. Eventually, we identified that hsa-miR-15a-3p might serve as a protective module against INH-induced liver injury by targeting *NAT2* in human liver cells and mouse model. Our results provided new clues to elucidate the epigenetic regulatory mechanisms concerning DILI.

## Materials and Methods

### Chemicals and Reagents

The hsa-miR-15a-3p mimics, miRNA negative control (NC), NC agomir, and mmu-miR-15a-3p agomir were purchased from Ribo Life Science (Guangzhou, China). All oligonucleotides and primers used in our study were obtained from Sangon Biotech (Shanghai, China), and their corresponding sequences were listed in Supplementary Table S1. All reporter gene constructs were produced by GENEray Biotech (Shanghai, China). Rabbit anti-human antibody against NAT2 protein was purchased from Gene Tex (Irvine, CA), rabbit anti-mouse antibody against NAT2 protein was purchased from ABclonal (Wuhan, China), rabbit anti-human antibody against β-actin was purchased from Servicebio (Wuhan, China) and horseradish peroxidase (HRP)-conjugated secondary antibody was obtained from BOSTER (Wuhan, China). Cell counting kit 8 was purchased from Dojindo (Kumamoto, Japan). Dual-Luciferase Reporter 1000 Assay System was purchased from Promega (Madison, WI). QuantiTect Reverse Transcription kit, miScript II RT Kit, and Quanti Fast TB Green RT-PCR kit were obtained from Qiagen (Valencia, CA). LightShift chemiluminescent RNA EMSA kits, Revert Aid First Strand cDNA Synthesis Kit, TRIzol Reagent, BCA Protein Assay Kit, SuperSignal West Femto Maximum Sensitivity Substrate, and Lipofectamine 2000 reagent were purchased from Thermo Fisher Scientific (Waltham, MA). Radioimmunoprecipitation assay (RIPA) buffer was purchased from Beyotime Biotechnology (Shanghai, China). Isoniazid (INH) was purchased from Sigma-Aldrich (St. Louis, MO). Alanine aminotransferase (ALT), aspartate aminotransferase (AST), and lactate dehydrogenase (LDH) kits were obtained from Nanjing Jiancheng (Nanjing, China). All other reagents were of analytical grade in this study.

### *In Silico* Analysis

The miRTar database (http://mirtar.mbc.nctu.edu.tw/human/) was used to screen potential miRNA binding sites located in the 3′-UTR of *NAT2* gene. The free energy of miRNA:mRNA duplexes was calculated by RNAhybrid program (http://bibiserv2.cebitec.uni-bielefeld.de/rnahybrid). Pearson’s correlation analysis (http://www.socscistatistics.com/tests/pearson/) was performed to evaluate the correlations between *NAT2* mRNA and each of the candidate miRNAs, based on the RNA levels in human liver samples (419 cases) obtained from TCGA (The Cancer Genome Atlas) database. Chemical names interacting with *NAT2* was obtained from the Comparative Toxicogenomics Database (CTD, http://ctd.mdibl.org) and ChemiRs Database (http://omics.biol.ntnu.edu.tw/ChemiRs).

### Cell Culture, Cell Transfection, and Chemical Treatment

The liver cancer cell lines Huh7 and HepG2, and human embryonic kidney cells HEK293T were obtained from the American Type Culture Collection (ATCC, Manassas, VA), respectively. The cells were incubated in Dulbecco’s modified Eagle’s medium (DMEM) containing 10% fetal bovine serum (FBS), 0.1 mg/ml streptomycin, and 100 U/mL penicillin (HepG2 cell culture requires 1% nonessential amino acids) at 37°C in a humidified 5% CO_2_ atmosphere.

The hsa-miR-15a-3p mimics and miRNA NC were transiently transfected into Huh7 and HepG2 cells by Lipofectamine 2000. After incubation for 48 h, cells were harvested for further analysis. Each experiment was performed three times independently.

The molecular weight of INH was 137.14 g/mol. According to its molecular weight, INH was dissolved in PBS to make the concentration of mother liquor 500 mM. The prepared solution was dropped into the cell medium to reach the working concentration of INH 10, 20, and 40 mM. The cells were incubated for 24 h and collected for subsequent experiments.

### Fluorescence-Based RNA Electrophoretic Mobility Shift Assay

FREMSA is an *in vitro* assay that could visually and accurately observe miRNA binding to its target RNA (Yu et al., 2020). The oligonucleotides for hsa-miR-15a-3p were synthesized and 5′-labeled with *cy5.5TM dye*. The 3′-UTR of *NAT2* were 5′-labeled with *IRDye* ^*®*^ *800 dye*. The unlabeled oligonucleotides, including the miRNA negative control (cold-NC) and hsa-miR-15a-3p (cold-miR-15a-3p), were used in competition assays. The oligonucleotide sequences are shown in the Supplementary Table S1. Briefly, the reaction system (containing 1 × binding buffer, 5% glycerin, 200 mM KCl, 100 mM MgCl_2_, and 200 nmol synthesized oligonucleotides) was incubated at room temperature to form miRNA:mRNA duplexes, separated by 12% PAGE electrophoresis at 4°C, and then detected by Odyssey CLx Infrared Imaging System (LI-COR Biosciences, Lincoln, NE, United States). Experiments were performed at least three times independently.

### Dual-Luciferase Reporter Gene Assay

The wild type 3′-UTR sequence of *NAT2* that harboring the targeting site of hsa-miR-15a-3p, and the mutated sequences that abolishing the targeting site of hsa-miR-15a-3p, were inserted into pmir-Glo vector, respectively. The constructed plasmids were co-transfected into the HEK293T cell using Lipofectamine 2000 according to the manufacturer’s instructions, together with hsa-miR-15a-3p mimics or miR-NC. At 48 h after transfection, the luciferase activity was detected using Dual-Luciferase Reporter 1000 Assay System. The relative activity of *Firefly* luciferase was normalized to *Renilla* luciferase activity. Each experiment was performed three times independently.

### Quantitative Real-Time Polymerase Chain Reaction

Trizol reagent was used to extract total RNAs from Huh7 and HepG2 cells. The mRNAs were reversely transcribed to complementary DNA by the Reverse Transcriptase Kit. The miRNAs were reversely transcribed by the miScript II RT Kit. The real-time PCR analysis was conducted using the TB Green Mixture in the LightCycler® 480 Detection System (Roche, Basel, Switzerland). For primer sequences, Supplementary Table S1. The relative *NAT2* mRNA (Genbank: NM_000015) and miR-15a-3p levels were normalized to β-actin and U6, respectively, through 2^−ΔΔCt^ method. Each experiment was performed three times independently.

### ALT, AST, LDH Assays

ALT, AST, and LDH kits were bought from Nanjing Jiancheng Bioengineering Institute. The operation procedure is according to the reagent manufacturer’s instructions.

### Western Blot Assay

Cells were lysed in RIPA buffer on ice. Total proteins were measured by BCA Protein Assay Kit according to the manufacturer’s protocol. The protein samples were separated by SDS-PAGE and transferred to a PVDF membrane. The harvested membrane was then incubated with antibodies against NAT2 and β-actin, respectively. After incubation with the HRP-conjugated secondary antibody, SuperSignal™ West Femto Maximum Sensitivity Substrate was used to detect the proteins. Each sample was evaluated for three independent experiments.

### Animals and Treatments

Eight-week-old male C57BL/6JNifdc mice in this study were purchased from Beijing Vital River Laboratory Animal Technology Co., Ltd. Mice were fed under standard pathogen free (SPF) conditions, 24°C ± 2°C, relative humidity 40–70%. During the experiment, the mice had free access to water and basic feed, and were weighed daily.

The mice model of chemical-induced liver injury was established by intragastric administration of INH. The permissible volume of mice gavage per 10g was 100ul. In people, the recommended dose is 5 mg/kg daily up to a maximum of 300 mg in most patients. The mice were given 45–135 mg/kg·d. Thus, the intragastric concentration of INH was set at 45 mg/kg·d, 90 mg/kg·d and 135 mg/kg·d (dissolved in normal saline), and the control group was intragastrically injected with appropriate volume of normal saline according to weight.

The first batch of mice was randomly assigned to four groups (6 C57BL/6JNifdc mice in each group), including the saline group, 45 mg/kg·d INH group, 90 mg/kg·d INH group, and 135 mg/kg·d INH group, according to the intragastric administration of a different dosage of INH. The second batch of mice was assigned to the miR-NC group (mice treated with INH and NC agomir) and miR-15a-3p group (mice treated with INH and mmu-miR-15a-3p agomir) (6 C57BL/6JNifdc mice in each group). Both groups of mice received 90 mg/kg·d of INH, to develop the mouse model of INH-induced liver injury. The cholesterol conjugated mmu-miR-15a-3p agomir and NC agomir were dissolved in PBS and injected into tail vein (5 nmol/time) every 3 days, respectively.

Upon desired timepoints (one or 2 weeks, *n* = 3), the animals were sacrificed, and serum and liver samples were collected, respectively. ALT, AST, and LDH were tested using serum samples. One portion of liver sample was fixed in 4% formaldehyde for pathological sections, and the other portion was stored in liquid nitrogen for RNA and protein extraction of liver tissue.

The mice were anesthetized by intraperitoneal injection of 1% pentobarbital sodium. The animal study was reviewed and approved by the Qingdao University Animal Care and Use Committee (No.20200827C576J701118002).

### Histological Analysis

As mentioned above, liver tissue was preserved in 4% formalin, embedded in paraffin, serially sectioned (6 μm), and stained with hematoxylin and eosin (H&E). The histological changes in liver tissue were measured using a light microscope.

### Statistical Analysis

SPSS and Prism were used for statistical analysis of the biological data results, which were presented as mean ± SD in the bar graphs. The expression levels of miRNA and NAT2 in liver tissue of TCGA database were used to calculate the correlation between the gene *NAT2* and miRNA by *Pearson* correlation analysis. One-way analysis of variance (ANOVA) was used to test the differences between subgroups in double-luciferase reporter gene, qRT-PCR, enzyme activity assay and pathological score among subgroups, respectively. And *LSD* test was used for comparison between the two groups. *p* < 0.05 were considered as statistically significant, and each experiment was performed at least three times.

## Results

### Hsa-miR-15a-3p Potentially Targets *NAT2* Transcript

The miRNAs that potentially targeting the 3′-UTR of *NAT2* were predicted using the miRTar.human database. As shown in Table 1, four mature miRNAs including hsa-miR-15a-3p, hsa-miR-628-5p, hsa-miR-1262, and hsa-miR-3132 were considered as potential epigenetic factors of *NAT2*. We further calculated the correlations between the miRNA expression and RNA levels of *NAT2* in 419 liver samples obtained from the TCGA database, and found a negative correlation (*r* = –0.192, *p* < 0.001) between hsa-miR-15a-3p and *NAT2* (NM_000015). No significant correlation was observed between the RNA levels of the other three candidate miRNAs and *NAT2* (data not shown). We therefore selected hsa-miR-15a-3p for further functional experiments.

**TABLE 1:** miRNAs potentially targeting *NAT2* gene.

Gene symbol	Transcript	miRNA symbol	Targeting position	Free energy (kcal/mol)^a^
*NAT2*	NM_000015	hsa-miR-15a-3p	1,189–1,210	–26.0^b^
*NAT2*	NM_000015	hsa-miR-628-5p	1,106–1,126	–20.8
*NAT2*	NM_000015	hsa-miR-1262	999–1,020	–22.1
*NAT2*	NM_000015	hsa-miR-3132	1,045–1,066	–29.9

a Calculated by the RNAhybrid program.

b r = –0.192, p < 0.001.

### Hsa-miR-15a-3p Interacted With NAT2 Transcript *in vitro*

**FIGURE 1:**
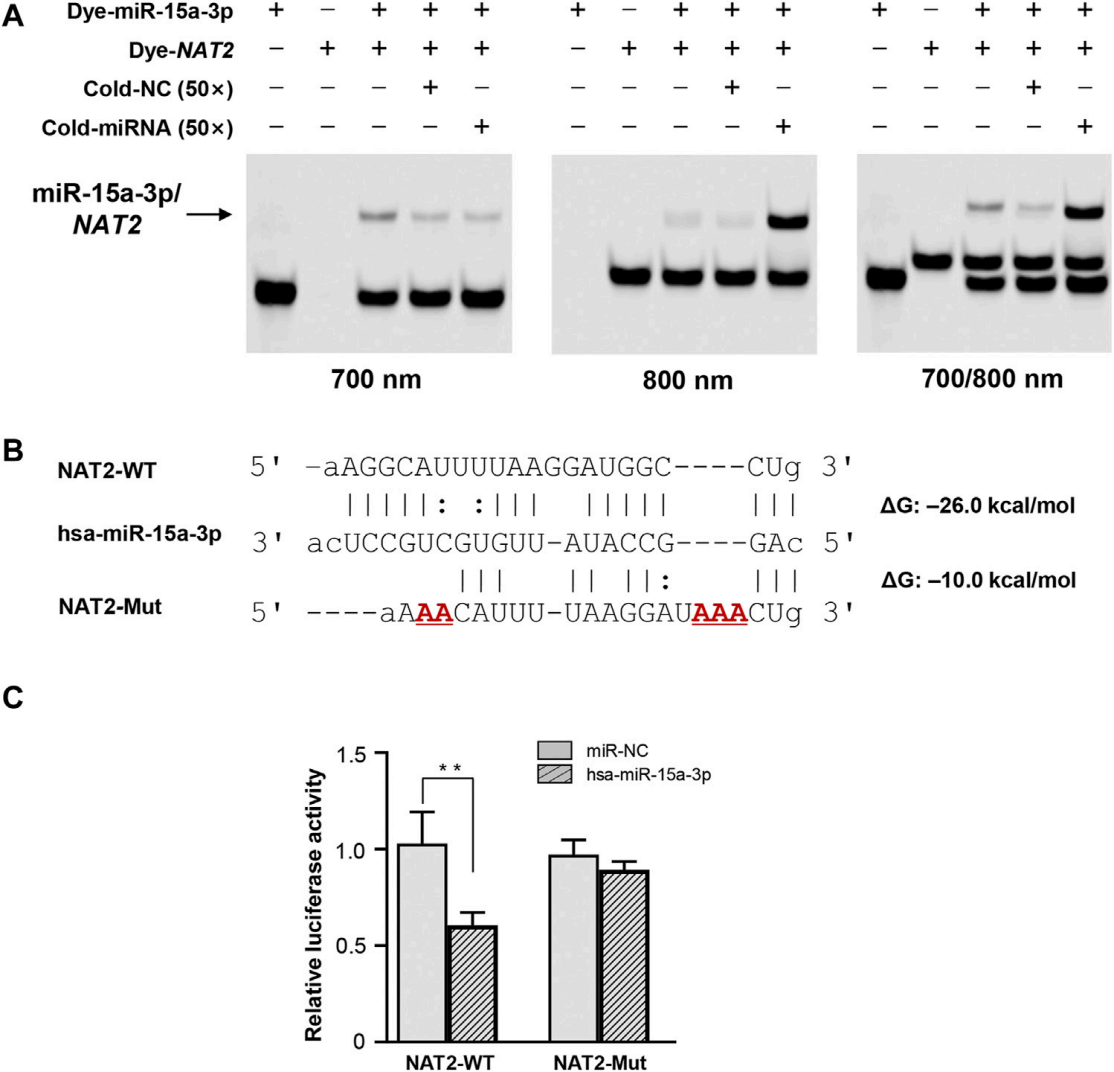
The hsa-miR-15a-3p oligonucleotides interacted with *NAT2* mRNA oligonucleotides *in vitro* and inhibited luciferase reporter gene expression. **(A)** *Lanes* 1 and 2 represented the hsa-miR-15a-3p and *NAT2* RNA oligonucleotides, respectively; *lane* 3 represented the formation of miRNA:mRNA complex; *lane* 4 represented the formation of miRNA:mRNA complex in the presence of excess unlabeled nonspecific competitors; *lane* 5 represented the formation of miRNA:mRNA complex in the presence of excess labeled specific competitors. NC, nonspecific competitor. **(B)** Free energy analyses of miRNA: mRNA duplex formed by hsa-miR-15a-3p and wild or mutated response elements in 3′-UTRs of *NAT2* (the RNAhybrid algorithm). **(C)** Constructs containing the wild or mutant type sequence of the core 3′-UTRs of *NAT2* gene were transiently transfected into HEK293T cells, respectively, together with 50 nmol/L hsa-miR-15a-3p mimic or miRNA negative control. Cells were harvested 48 h after transfection. The data represented three independent experiments, each in triplicate reactions, were shown as mean ±SD. **p < 0.01.

FREMSA was conducted to detect the formation of miRNA:mRNA duplexes *in vitro*. As shown in Figure 1A, dye-miR-15a-3p oligonucleotides could interact with dye-*NAT2* oligonucleotides to form a stable miRNA:mRNA complex that showed a significant mobility shift (*lane 3*). Excess unlabeled NC oligonucleotides (cold-NC) failed to completely abolish the miRNA:mRNA complex (*lane 4*), indicating its high stability. Further, excess unlabeled miR-15a-3p oligonucleotides could competitively weaken the miRNA:mRNA complex that formed by dye-miR-15a-3p and dye-*NAT2* oligonucleotides, and then produced more miRNA:mRNA complex with dye-*NAT2* oligonucleotides (*lane 5*).

### Hsa-miR-15a-3p Suppressed Luciferase Activity Driven by *NAT2* 3′-UTR

The wild type and mutated 3′-UTR sequence of *NAT2* that harboring the targeting site of hsa-miR-15a-3p was subcloned into luciferase reporter gene vector (pmir-Glo), respectively (Figure 1B). The constructed vectors were then co-transfected into HEK293T cells with hsa-miR-15a-3p mimics or miR-NC. As shown in Figure 1C, exogeneous hsa-miR-15a-3p significantly reduced the luciferase activity produced by the reporter gene plasmid containing the wild type 3′-UTR of *NAT2* (42.3%, *p* < 0.01), compared to that in cells treated with miR-NC. Further, the luciferase activity driven by the mutant plasmid that abolished the target site of hsa-miR-15a-3p, failed to be suppressed by hsa-miR-15a-3p, indicating a sequence specificity of hsa-miR-15a-3p binding to *NAT2* transcript.

### Exogeneous Hsa-miR-15a-3p Down-Regulated Endogenous *NAT2* Expression

**FIGURE 2:**
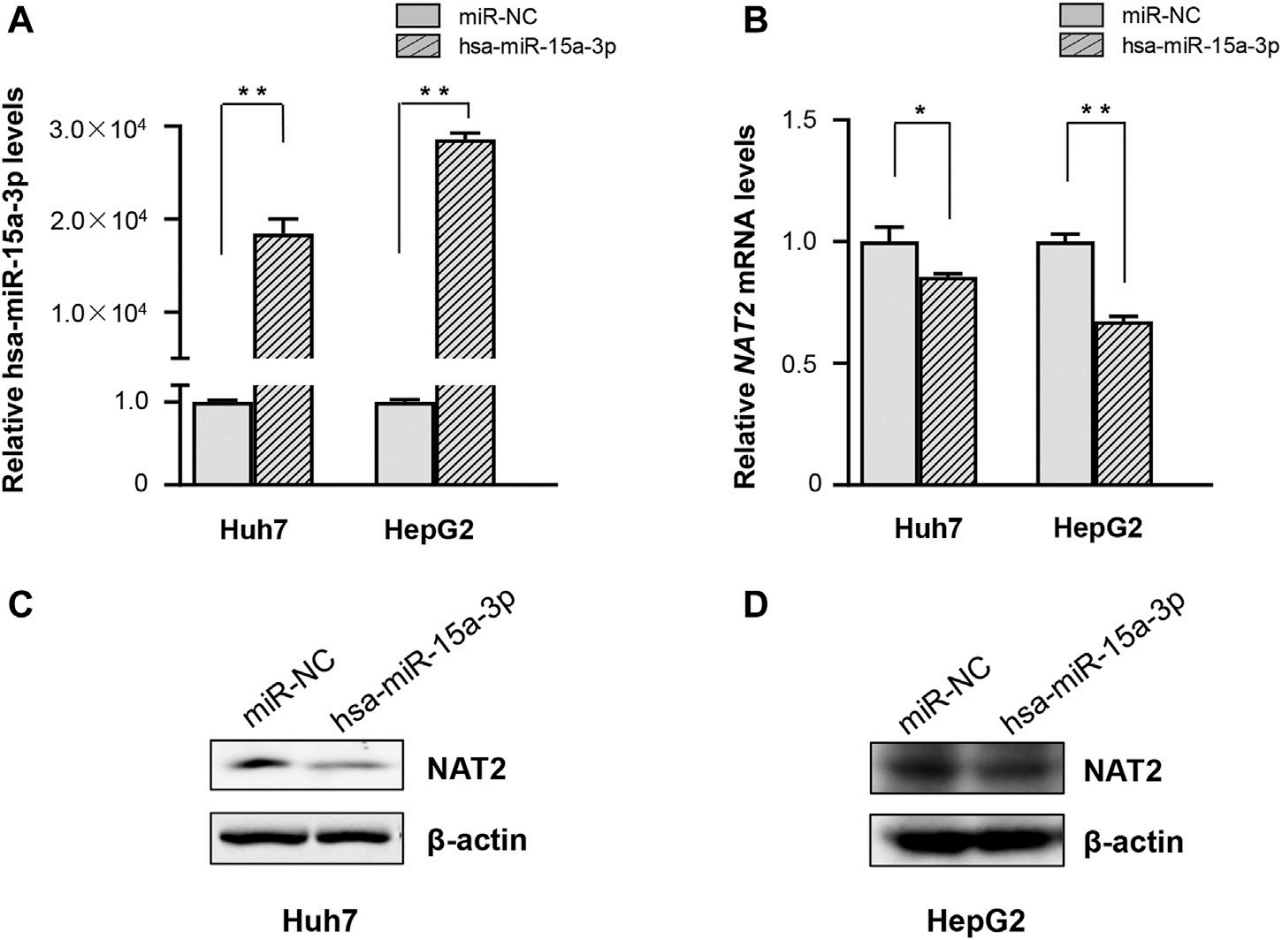
The hsa-miR-15a-3p inhibited endogenous *NAT2* expression in Huh7 and HepG2 cells. hsa-miR-15a-3p mimics or miRNA NC (both at the final concentration of 20 μmol/L) was transfected into Huh7 and HepG2 cells, respectively. **(A)** hsa-miR-15a-3p levels in Huh7 and HepG2 cells after transfection with hsa-miR-15a-3p mimics. **(B)** *NAT2* expression in Huh7 and HepG2 cells after transfection with hsa-miR-15a-3p mimics. **(C,D)** Protein levels of *NAT2* in Huh7 and HepG2 cells after transfection with exogeneous hsa-miR-15a-3p mimics. Data represented three independent experiments and were shown as mean ± SD. The fold changes of miRNA, mRNAs, and proteins when transfected with exogenous hsa-miR-15a-3p mimics were calculated based on each of their levels when transfected with miRNA negative control (miR-NC), which was defined as 1. *p < 0.05; **p < 0.01.

Subsequently, hsa-miR-15a-3p mimics and miRNA-NC were transfected into Huh7 and HepG2 cells, respectively. Under our experimental conditions, transfection of hsa-miR-15a-3p mimics significantly elevated endogenous hsa-miR-15a-3p levels (Figure 2A), and substantially reduced the mRNA levels of *NAT2* in both Huh7 and HepG2 cells (Figure 2B, by 14.4% in Huh7 cells, *p* < 0.05 and by 32.6% in HepG2 cells, *p* < 0.01), compared to the control group. Similarly, western blot assays showed that endogenous NAT2 protein levels were significantly reduced in hepatoma cells treated with exogenous hsa-miR-15a-3p mimics, compared to the control group (Figures 2C,D).

### Exogeneous Hsa-miR-15a-3p Inhibited INH-Induced *NAT2* Overexpression

**FIGURE 3:**
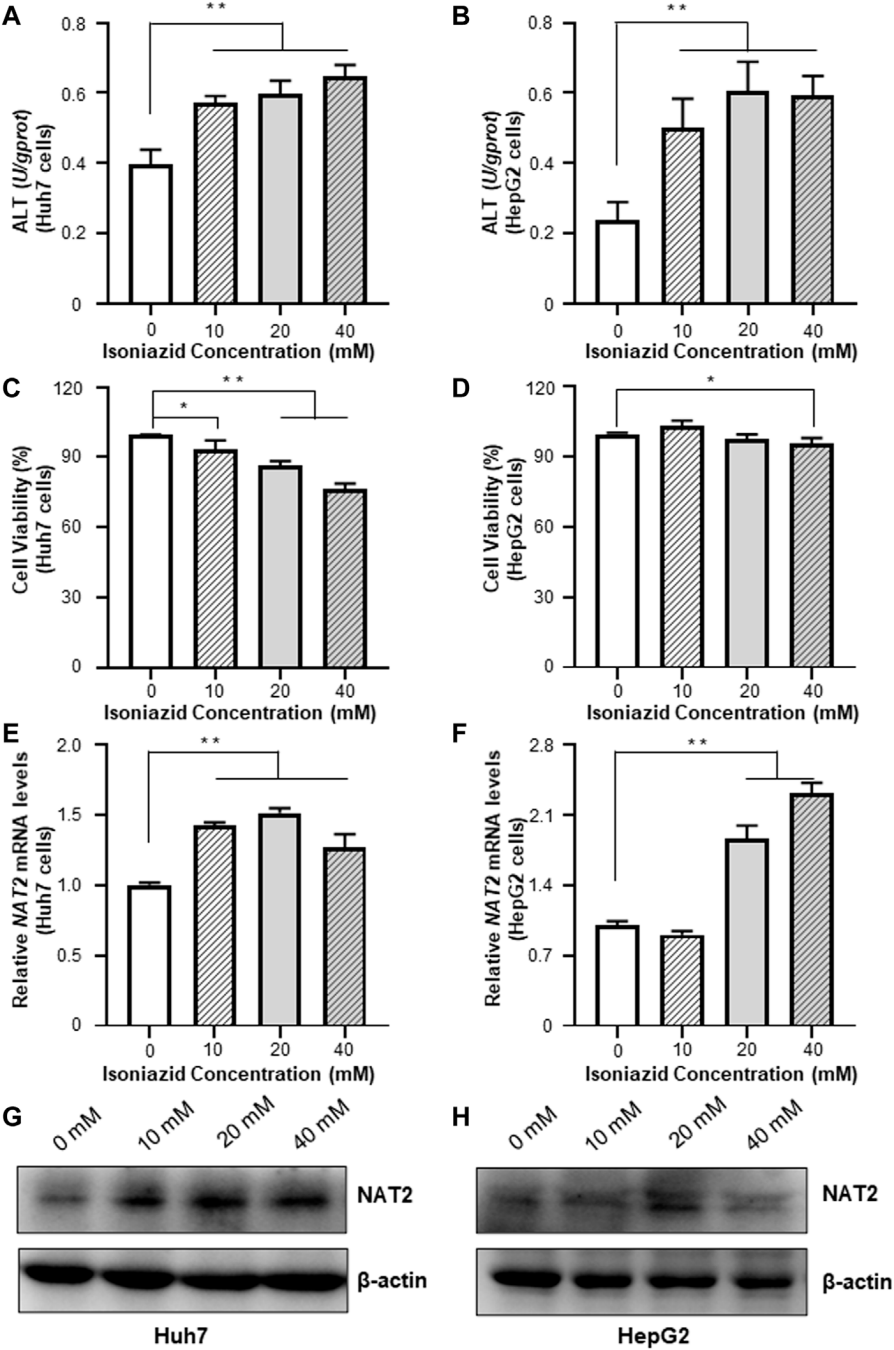
Effects of INH on the ALT enzyme activities and *NAT2* expression of Huh7 and HepG2 cells. **(A,B)** Changes of ALT enzyme activities in Huh7 and HepG2 cells treated with INH. **(C,D)** Changes of cell viability in Huh7 and HepG2 cells treated with INH. **(E,F)** Exposure to INH at different working concentrations caused the expression of *NAT2* mRNA levels in Huh7 and HepG2 cells. **(G,H)** Exposure to INH at different working concentrations caused the expression of *NAT2* protein levels in Huh7 and HepG2 cells. Data represented three independent experiments and were shown as mean ± SD. *p < 0.05; **p < 0.01.

We treated the Huh7 and HepG2 cells with different dosages of INH, and evaluated the resultant cellular toxicity via testing the ALT levels. As shown in Figures 3A,B, the ALT activity was significantly elevated (by 44.5, 51.5, and 64.1% in Huh7 cells; 1.1-fold, 1.6-fold, and 1.5-fold in HepG2 cells, all *p* < 0.01), and positively associated with the increased concentration of INH. The cell viability decreased with the increase of INH concentration in Huh7 cells, but the cell viability decreased slightly in HepG2 cells due to the agglomeration growth (Figures 3C,D). In addition, qRT-PCR results revealed that INH treatment increased endogenous RNA of *NAT2* gene (Figures 3E,F). However, in terms of NAT2 protein level, it was increased only in Huh7 cells after INH treatment, while this phenomenon was not obvious in HepG2 cells (Figures 3G,H).

**FIGURE 4:**
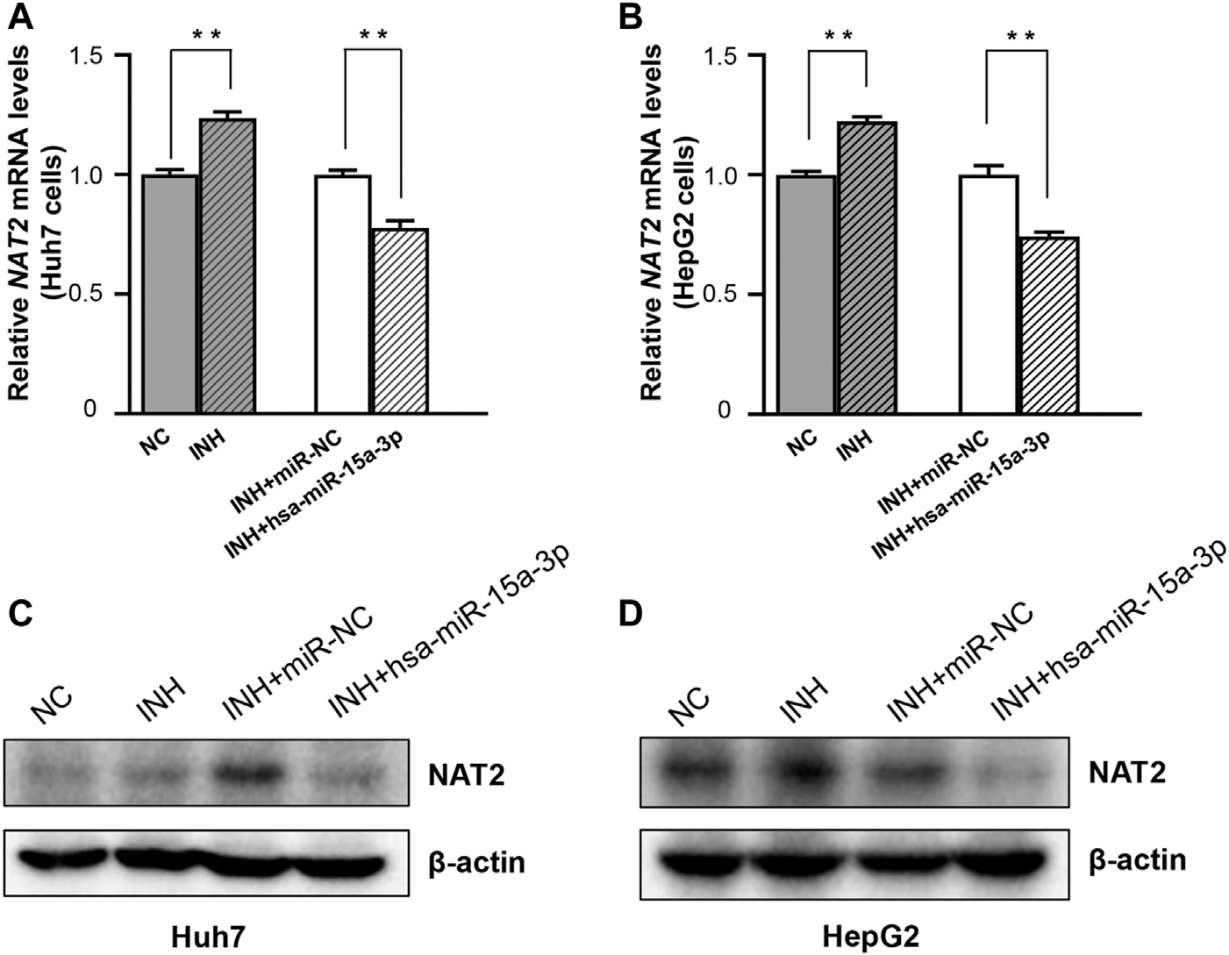
The hsa-miR-15a-3p suppressed INH-induced *NAT2* expression in Huh7 and HepG2 cells. **(A,B)** Effect of exogenous hsa-miR-15a-3p on *NAT2* mRNA levels in Huh7 and HepG2 cells exposed to INH (final concentration: 40 mM). **(C,D)** Effect of exogenous hsa-miR-15a-3p on *NAT2* protein levels in Huh7 and HepG2 cells exposed to INH (final concentration: 40 mM). Data represented three independent experiments and were shown as mean ± SD. The fold changes of mRNAs, and proteins in INH treatment group (INH) were calculated by defining their levels in the untreated group (NC) as 1. Similarly, the fold changes of mRNAs, and proteins in the group of INH treatment together with miR-15a-3p transfection (INH + hsa-miR-15a-3p) were calculated by defining their levels in the group of INH treatment together with miRNA negative control transfection (INH + miR-NC) as 1. **p < 0.01.

Further, both Huh7 and HepG2 cells were transfected with hsa-miR-15a-3p mimics and miRNA-NC, respectively, and then exposed to INH at a final concentration of 40 mM. As shown in Figure 4, qRT-PCR results showed that exogeneous hsa-miR-15a-3p was able to inhibit the INH-induced mRNA elevation of *NAT2* gene (by 22.3% in Huh7 cells and 25.8% in HepG2 cells, respectively, both *p* < 0.01), compared to each NC group (Figures 4A,B). Similar inhibitory effects were also observed in the protein levels of NAT2 in Huh7 cells (*lane 3* vs. *lane 4*, Figure 4C). Although we did not find the obvious increase of NAT2 protein induced by INH previously, we found the inhibitory effect of hsa-miR-15a-3p on NAT2 after INH treatment (*lane 3* vs. *lane 4*, Figure 4D).

### MiR-15a-3p Protected Against INH-Induced Liver Injury in C57BL/6JNifdc Mice

**FIGURE 5:**
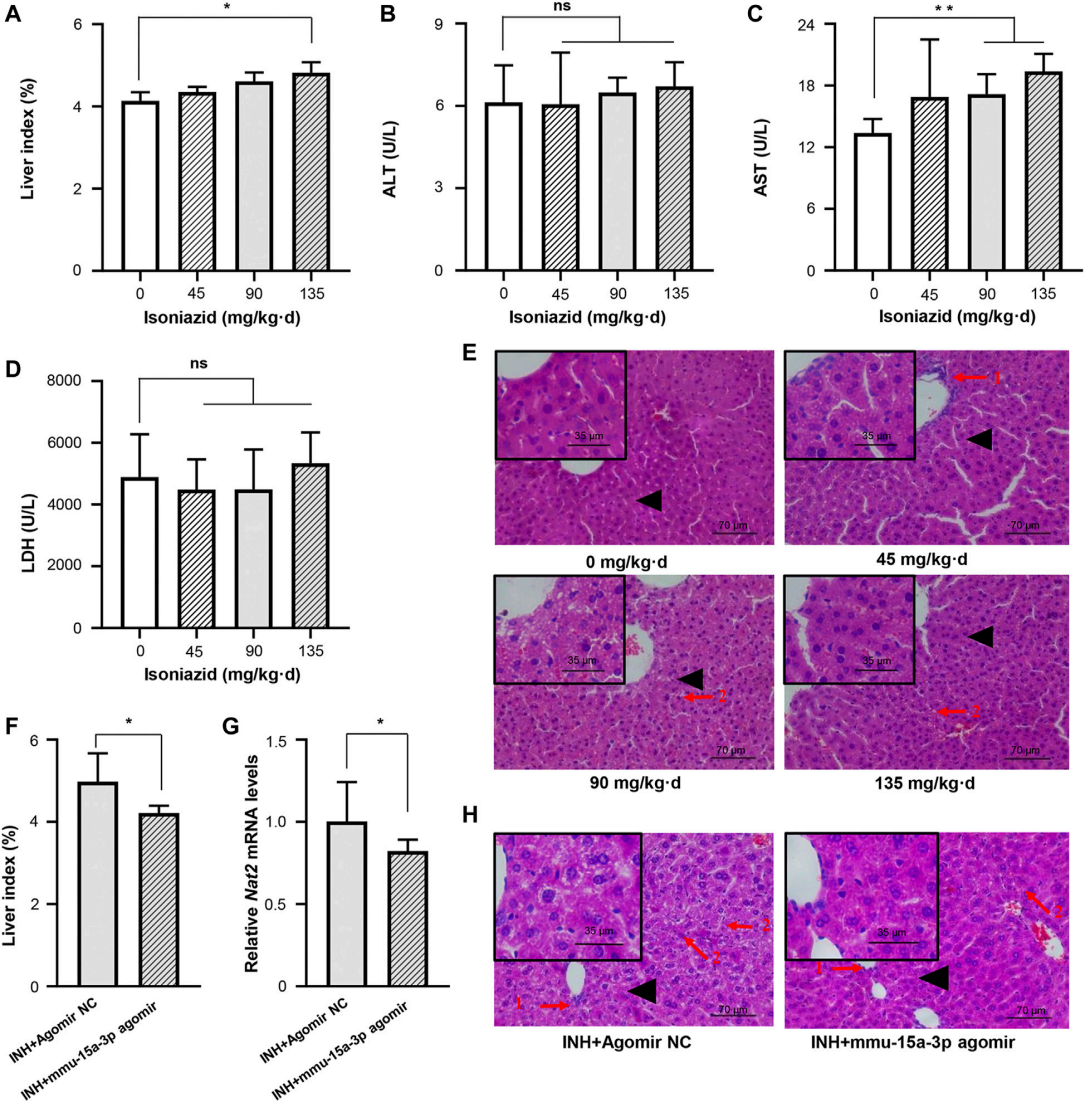
Liver injury in mice induced by INH was alleviated by mmu-miR-15a-3p agomir. Mice were administrated with normal saline, 45 mg/kg INH, 90 mg/kg INH, and 135 mg/kg INH for 14 days (*n* = 3). After the last administration for 18 h, mice were sacrificed and blood was collected. **(A)** The liver index of each group was measured. **(B)** Serum ALT was determined. **(C)** Serum AST was determined. **(D)** Serum LDH was determined. **(E)** Liver histopathological examination was performed by using H&E staining (X400). **(F)** After the intervention of mmu-miR-15a-3p agomir, the changes of liver index in liver injured mice were measured (*n* = 6). **(G)** The mRNA expression level of *Nat2* in liver tissues was quantified. And **(H)** Liver histopathological examination was performed by using H&E staining (X400). Arrow 1: Inflammatory cell infiltration; Arrow 2: Microvesicular steatosis; Black triangle: local magnification position. *p < 0.05; **p < 0.01.

Accumulating evidences proved that INH was able to induce liver injury, but seldom observed the significant dysregulation of serum indicators including ALT, AST, and LDH (Metushi et al., 2016; Lian et al., 2017; Ci et al., 2020). In this study, C57BL/6JNifdc mice received intragastric administrations of 0, 45, 90, and 135 mg/kg·d of INH for one or 2 weeks, respectively. Compared to the control group, both one-week and two-week INH treatment increased the liver index of mice (liver index = liver wet weight/body weight × 100%), in a dose-dependent manner (Figure 5A and Supplementary Figure S1A). Consistent with the studies mentioned above, neither ALT nor LDH activity was increased by INH treatment, this conclusion is consistent with the articles on isoniazid-induced liver injury; however, we indeed observed that AST activity was significantly increased in the 90 and 135 mg/kg·d INH group, compared to the control group (Figures 5B–D and Supplementary Figures S1B–S1D). Histopathological change was considered as a pivotal criterion to judge the occurrence of liver injury. H&E staining was used after 1 or 2 weeks of exposure, normal liver cell morphology and intact cytoplasm were observed in the control group without necrosis or inflammation. In the 45, 90 and 135 mg/kg·d INH groups, the mice showed significant cell enlargement, inflammatory cell infiltration, and microvesicular steatosis (nucleus centered). Compared to the mice received one-week INH treatment, a wider range of hepatic fatty lesions was identified in that received two-week INH treatment (Figure 5E and Supplementary Figure S1E). Eventually, two-week intragastric administration of 90 mg/kg·d of INH was selected to create the mouse model of INH-induced liver injury.

We injected mmu-miR-15a-3p agomir into this mouse model and evaluated the protective effects of miR-15a-3p against INH-induced liver injury *in vivo*. As shown in Figure 5F, the liver index was significantly reduced after the intervention of mmu-miR-15a-3p agomir, compared to the control group. Pathological results provided that obviously cell enlargement, more small lipid droplets and inflammatory cytometry were observed in control group, however, the degree and range of liver steatosis were significantly reduced after the intervention of mmu-miR-15a-3p agomir (Figure 5H **)**. More solid evidence illustrated that exogeneous miR-15a was able to protect cells against INH-induced liver injury. In addition, we observed that injection of mmu-miR-15a-3p agomir significantly decreased *Nat2* transcripts (18%, *p* < 0.05; Figure 5G). Due to the specificity and stability of the anti-mouse polyclonal antibody Nat2, stable expression of Nat2 protein in mouse liver was not detected in this study.

## Discussion

Understanding the regulations of DMEs is critical in making medical decisions, improving drug use efficiency and avoiding adverse reactions due to drug abuse or misuse. Some studies have revealed that NAT2, CYP2E1*,* and other DMEs are involved in the bioconversion of INH (Ben Fredj et al., 2017). Moreover, the polymorphism of *the NAT2* genotype has been reported as directly associated with liver injury induced by INH (Xiang et al., 2014; Zhang et al., 2018). In this study, we investigated the role of miRNA in regulating the expression of *NAT2* with and without INH exposure, and identified hsa-miR-15a-3p as a protective molecule in INH-induced liver injury.

NATs is phase II of drug metabolizing enzymes in most mammals (Jancova et al., 2010). Human NAT has two subtypes, *NAT1* and *NAT2*. They differ greatly in terms of tissue distribution and biological function. *NAT1* is expressed in most tissues and is responsible for catalyzation of acetylation of amino salicylic acid and para-aminobenzoic acid, while *NAT2*, also known as aromatic amine *N*-acetyltransferase, mainly catalyzes the acetylation and transferring process of aromatic amine (Hickman et al., 1998; Jancova et al., 2010), which takes place in the liver and intestines. Earlier studies have shown that catalytic transfer of acetyl groups from acetyl CoA to nitrogen atoms of INH in turn inactivated the compound (Sim et al., 2000). It was found early that the functional variation of *NAT2* was related to the diversity of drug reactions (Meisel, 2002), the acetylation of INH being one example (Dickinson et al., 1981; Sim et al., 2014). All above studies confirmed the significance of investigating the regulation of *NAT2* in understanding INH metabolism and related liver injury.

At present, nearly a hundred miRNA sequence prediction tools have been invented to accurately identify miRNA targets, and experimental methods are generally considered for further characterization of the functions of miRNAs in the biological process (Riffo-Campos et al., 2016). Unfortunately, *in vitro* experiments often failed to agree with the predicted interactions between the miRNA and mRNA target, possibly due to the limitations of prediction using pure algorithms as these algorithmic tools allow users to customize free energy thresholds, *p* values, and the location and length of seed regions (Thomas et al., 2010; Li and Zhang, 2015). In the past few years, we have predicted and successfully demonstrated the roles of miRNAs in regulating multiple DMETs by integrating bioinformatics analysis with *in vivo* and *in vitro* methods (Zeng et al., 2017; Knox et al., 2018; Yu et al., 2018). In this study, we first screened candidate miRNAs *in silico* and found that 4 miRNAs may interact with the 3′-UTR of *NAT2*. Among them, hsa-miR-15a-3p was selected for functional validation, because of its high free energy to target the nucleotide position 1,189–1,210 of the NM transcript (–26.0 kcal/mol) and negative correlation with *NAT2* expression in liver tissues.

Subsequently, FREMSA assays were performed to determine whether hsa-miR-15a-3p was able to interact with the 3′-UTR of *NAT2* directly. The results of FREMSA were consistent with *in silico* analysis under our experimental conditions. Transient transfection of hsa-miR-15a-3p in hepatoma cell lines (Huh7 and HepG2 cells) showed that hsa-miR-15a-3p suppressed *NAT2* production significantly. As expected in the *in silico* prediction, hsa-miR-15a-3p interacted with *NAT2* both *in vitro* and *in vivo*; Also, hsa-miR-15a-3p inhibited INH-induced *NAT2* expression and therefore, INH-induced liver toxicity, both in liver cell assays and mouse model.

Until now, only one study reported the miRNA targeting *NAT2* gene, in which miR-217 was observed to suppress proliferation and promote apoptosis by binding to *NAT2* transcript in the liver cells from rat model with CCl4-induced liver injury (Yang et al., 2019). In our study, miR-217 was not indicated in the prediction. One plausible reason could be that the free energy of miR-217 binding to *NAT2* transcript (NM_000015) is –13.6 kcal/mol, which failed to reach the selection threshold (–20.0 kcal/mol) applied in this study. As to miR-15a-3p, most studies focused on its roles in tumor initiation and progression. For example, Fan et al. and Wang et al. observed that miR-15a-3p inhibited the growth and invasion in ovarian and gastric cancer, respectively, by targeting *Twist1* (Fan et al., 2019; Wang et al., 2019). To our knowledge, this is the first study that reports the regulatory role of miR-15a-3p in drug safety or toxicity.

Oxidative stress is the common physiological basis of many diseases (Schattenberg et al., 2004). In type 2 diabetes, exo-miR-15a release is increased, which can be absorbed by Müller cells and inhibit the PI3-kinase signaling pathway, leading to oxidative stress and apoptotic cell death (Kamalden et al., 2017). In acute lung injury, miR-15a deficiency can lead to apoptosis of lung epithelial cells in hyperoxia response by modulating internal and external apoptotic pathways (Cao et al., 2016). These functional results further suggest that miR-15a performs different cellular functions in response to specific stimuli in different cell types and may in fact act as a homeostasis factor that balances cell death and survival depending on specific cell types and conditions. Since these are defined in animal studies, this information can be used to determine whether the same mechanisms also apply to patients.

Another noteworthy issue in INH-induced liver injury is the imbalance of accumulation and depletion of AcHz. As mentioned earlier, AcHz is generated from INH under NAT2 catalyzation, then catalyzed either by NAT2, namely detoxification, or by CYP2E1 to form the ultimately toxic acetyl diazene, namely toxicological activation. The impact of acetylation rate of NAT2 in INH-induced liver injury is controversial. Some studies suggested faster NAT2 acetylator might yield more AcHz and consequently greater damage of the cells, while some others observed that faster NAT2 acetylator resulted in less damage due to higher elimination efficiency of AcHz by NAT2 (Saukkonen et al., 2006). To solve this puzzle, the expression or activity ratio between NAT2 and CYP2E1, rather than the individual enzyme activity or expression, should be used to predict the risk of INH-induced liver injury. In addition, the interactions among miRNAs and other types of non-coding RNAs that regulate *NAT2* and *CYP2E1*, respectively, remain to be elucidated in the future.

To sum up, we observed that hsa-miR-15a-3p down-regulated *NAT2* expression in hepatocytes by directly targeting the 3′-UTR, and further found hsa-miR-15a-3p suppressed IHN-induced *NAT2* expression and consequently alleviated liver toxicity as shown both in cell lines and the mouse model. Our results provided novel evidences supporting the regulatory roles of miRNAs on DMEs, and illustrated the potential roles of miRNAs as biomarkers for DILI.

## Data Availability

The original contributions presented in the study are included in the article/Supplementary Material, further inquiries can be directed to the corresponding author.

## Abbreviations

AcHz, acetylhydrazine; ALT, alanine aminotransferase; ANOVA, one-way analysis of variance; AST, aspartate aminotransferase; CTD, Comparative Toxicogenomics Database; DiAcHz, diacetyl-hydrazine; DILI, drug-induced liver injury; DMEM, Dulbecco’s modified Eagle’s medium; DMEs, drug metabolizing enzymes; DMETs, drug metabolizing enzymes and transporters; FBS, fetal bovine serum; FREMSA, fluorescent-based RNA electrophoretic mobility shift assay; GSTs, glutathione-*S*-transferases; HRP, horseradish peroxidase; INH, isoniazid; LDH, lactate dehydrogenase; miRNA, microRNA; NATs, *N*-Acetyltransferases; NAT2, *N*-acetyltransferase 2; NC, negative control; qRT-PCR, quantitative real-time polymerase chain reaction; RIPA, radioimmunoprecipitation assay; SPF, standard pathogen free; TCGA, The Cancer Genome Atlas; UGTs, UDP-glucuronosyltransferases; UTR, untranslated region.
