# Supplementary material for: miR-15a-3p Protects Against Isoniazid-Induced Liver Injury via Suppressing N-Acetyltransferase 2 Expression
Source: Front Mol Biosci. 2021 Nov 23;8:752072. doi: 10.3389/fmolb.2021.752072 (PMC8651391; doi:10.3389/fmolb.2021.752072)
Supplement: Supplementary file 1 [file DataSheet1.PDF]

# *Supplementary Material*

## Supplementary Tables

**Supplementary Table 1. Sequences of primers and oligonucleotides**

| **Name** | **Sequence (5'–3')** |
|---|---|
| ***qRT-PCR primers*** | |
| NAT2-F | GGTGGTGTCTCCAGGTCAATCA |
| NAT2-R | TGCAGGAGAAGGTGAACCATGC |
| β-actin-F | GGCACCCAGCACAATGAAG |
| β-actin-R | CCGATCCACACGGAGTACTTG |
| hsa-miR-15a-3p-F | GGCAGGCCATATTGTGCTG |
| hsa-miR-15a-3p-R | CAGTGCGTGTCGTGGAGT |
| U6-F | CTCGCTTCGGCAGCACA |
| U6-R | AACGCTTCACGAATTTGCGT |
| ***FREMSA oligonucleotides*** | |
| Dye-miR-15a-3p | CAGGCCAUAUUGUGCUGCCUCA |
| Dye-NAT2 | AAGGCAUUUUAAGGAUGGCCUG |
| Cold miR-NC | UCACAACCUCCUAGAAAGAGUAGA |

Supplementary Figures

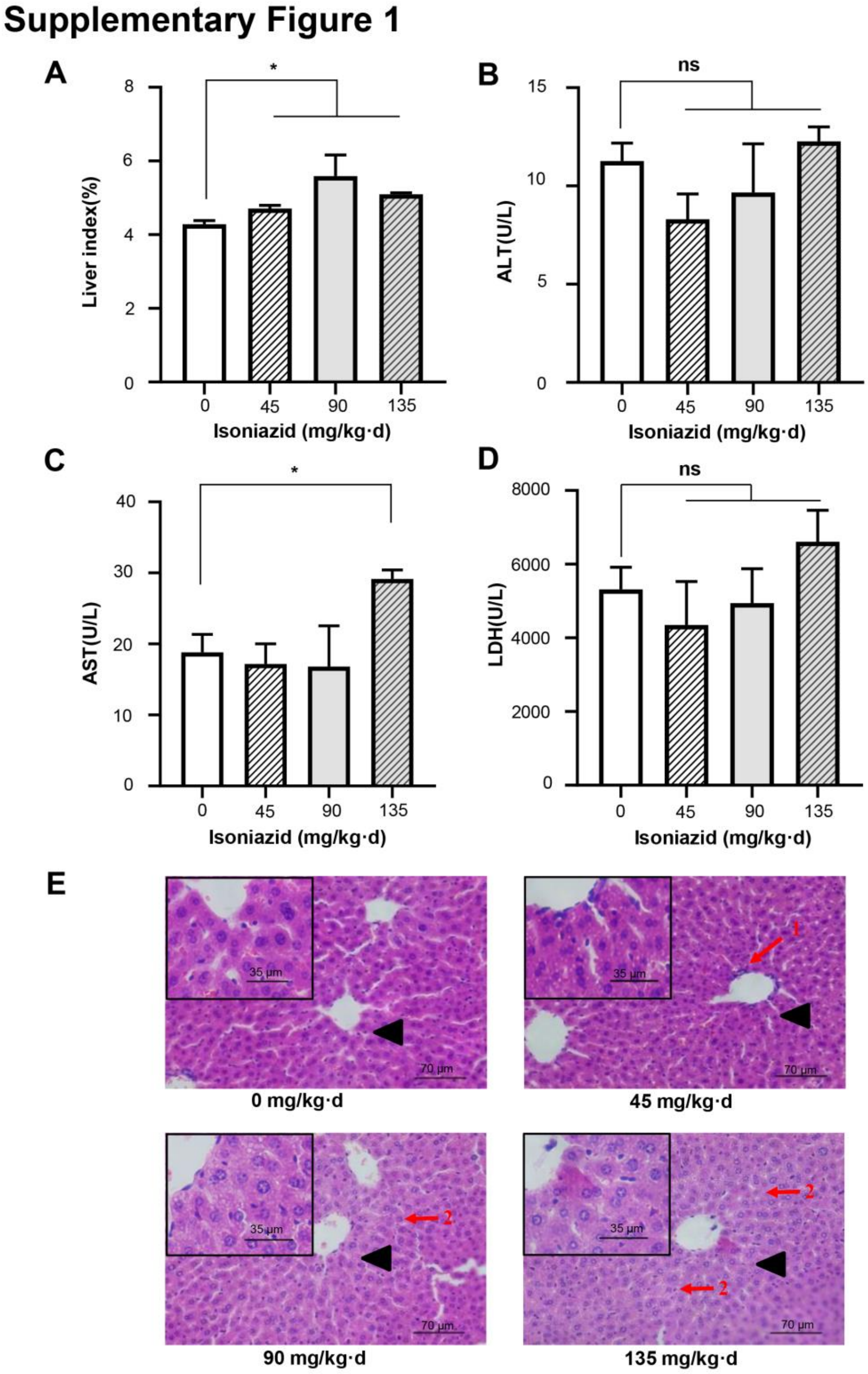


**Supplementary Figure 1. Liver injury in mice induced by INH was alleviated by mmu-miR-15a-3p agomir.** Mice were administrated with normal saline, 45 mg/kg INH, 90 mg/kg INH, and 135 mg/kg INH for 7 days (n=3). After the last administration for 18 h, mice were

sacrificed and blood was collected. **(A)** The liver index of each group was measured. **(B)** Serum ALT was determined. **(C)** Serum AST was determined. **(D)** Serum LDH was determined. **(E)** Liver histopathological examination was performed by using H&E staining (X400). Arrow 1: Inflammatory cell infiltration; Arrow 2: Microvesicular steatosis; Black triangle: local magnification position. $^{*}P < 0.05$.
